# Multi-omics of 34 colorectal cancer cell lines - a resource for biomedical studies

**DOI:** 10.1186/s12943-017-0691-y

**Published:** 2017-07-06

**Authors:** Kaja C. G. Berg, Peter W. Eide, Ina A. Eilertsen, Bjarne Johannessen, Jarle Bruun, Stine A. Danielsen, Merete Bjørnslett, Leonardo A. Meza-Zepeda, Mette Eknæs, Guro E. Lind, Ola Myklebost, Rolf I. Skotheim, Anita Sveen, Ragnhild A. Lothe

**Affiliations:** 1 Department of Molecular Oncology, Institute for Cancer Research & K.G.Jebsen Colorectal Cancer Research Centre, Oslo University Hospital, P.O.Box 4953 Nydalen, -0424 Oslo, NO Norway; 2 Center for Cancer Biomedicine, Institute for Clinical Medicine, University of Oslo, Oslo, Norway; 3 Norwegian Cancer Genomic Consortium, Oslo University Hospital, Oslo, Norway; 4 Department of Core Facilities and Department of Tumor Biology, Institute for Cancer Research, Oslo University Hospital, Oslo, Norway; 5 Department of Clinical Science, University of Bergen, Bergen, Norway

**Keywords:** Colorectal cancer cell lines, Consensus molecular subtypes, Copy number aberrations, Gene expression, Genomics, Methylation, Microsatellite instability, miRNA, Mutations, Protein expression

## Abstract

**Background:**

Colorectal cancer (CRC) cell lines are widely used pre-clinical model systems. Comprehensive insights into their molecular characteristics may improve model selection for biomedical studies.

**Methods:**

We have performed DNA, RNA and protein profiling of 34 cell lines, including (i) targeted deep sequencing (*n* = 612 genes) to detect single nucleotide variants and insertions/deletions; (ii) high resolution DNA copy number profiling; (iii) gene expression profiling at exon resolution; (iv) small RNA expression profiling by deep sequencing; and (v) protein expression analysis (*n* = 297 proteins) by reverse phase protein microarrays.

**Results:**

The cell lines were stratified according to the key molecular subtypes of CRC and data were integrated at two or more levels by computational analyses**.** We confirm that the frequencies and patterns of DNA aberrations are associated with genomic instability phenotypes and that the cell lines recapitulate the genomic profiles of primary carcinomas. Intrinsic expression subgroups are distinct from genomic subtypes, but consistent at the gene-, microRNA- and protein-level and dominated by two distinct clusters; *colon-like* cell lines characterized by expression of gastro-intestinal differentiation markers and *undifferentiated* cell lines showing upregulation of epithelial-mesenchymal transition and TGFβ signatures. This sample split was concordant with the gene expression-based consensus molecular subtypes of primary tumors. Approximately ¼ of the genes had consistent regulation at the DNA copy number and gene expression level, while expression of gene-protein pairs in general was strongly correlated. Consistent high-level DNA copy number amplification and outlier gene- and protein- expression was found for several oncogenes in individual cell lines, including *MYC* and *ERBB2*.

**Conclusions:**

This study expands the view of CRC cell lines as accurate molecular models of primary carcinomas, and we present integrated multi-level molecular data of 34 widely used cell lines in easily accessible formats, providing a resource for preclinical studies in CRC**.**

**Electronic supplementary material:**

The online version of this article (doi:10.1186/s12943-017-0691-y) contains supplementary material, which is available to authorized users.

## Background

Colorectal cancers (CRC) are molecularly heterogeneous and can be divided into clinically relevant subtypes associated with patient prognosis and treatment response. At the DNA level, this includes the genomic instability phenotypes microsatellite instability (MSI) and chromosomal instability (CIN), as well as the epigenomic CpG island methylator phenotype (CIMP). About 15% of primary CRCs have MSI, while the rest are microsatellite stable (MSS), most of which have the CIN phenotype. MSI tumors have errors in the mismatch repair machinery and display numerous single nucleotide variants (SNVs) and insertions/deletions (indels) [1]. CIN tumors typically display aneuploidy with structural and/or numerical aberrations, but the underlying cause(s) remains undetermined [2]. CIMP tumors overlap to a large extent with MSI and are characterized by widespread hypermethylation of CpG islands [3, 4].

At the transcriptional level, several classification schemes have identified biologically distinct subtypes of CRCs [5–7]. The recent identification of four consensus molecular subtypes (CMS) has provided evidence that the expression subtypes have clinical relevance independent of cancer stage [8]. Although several genomic aberrations associate with individual CMS groups, including MSI and hypermutation in CMS1 and oncogene amplification in CMS2, a potential genomic basis for the expression subtypes remains elusive. Integrative DNA, RNA and protein level analyses promise to improve our understanding of the biological and clinical importance of the evolving molecular classification of CRC.

CMS classification is heavily influenced by the tumor microenvironment, as demonstrated by strong expression of mesenchymal marker genes in the stroma of tumors of the stem-like/mesenchymal subtype CMS4 [9, 10]. However, all four CMS subtypes were recently demonstrated to be represented in in vitro model systems (Sveen et al., submitted), and cancer cell lines may therefore be used to identify the cancer cell intrinsic aberrations characteristic of the four CMS groups. Furthermore, genomic studies and drug sensitivity screening have demonstrated that CRC cell lines in general recapitulate the molecular alterations and pharmacogenomics of primary tumors [11–15]. Accordingly, improved molecular characterization of these in vitro model systems may further increase their value as preclinical models of CRC.

Here we present a resource of information for 34 CRC cell lines by multi-level data integration, including targeted deep sequencing, DNA copy numbers, gene expression, microRNA (miRNA) expression and protein expression. We describe consistent gene/pathway regulation across data types and associate this with known CRC subtypes. Each data set and data combination are presented in accessible tables and figures, emphasizing specific alterations of biological or clinical interest for further experimental studies.

## Methods

### Cell lines – Culturing, processing and analyses overview

**Fig. 1:**
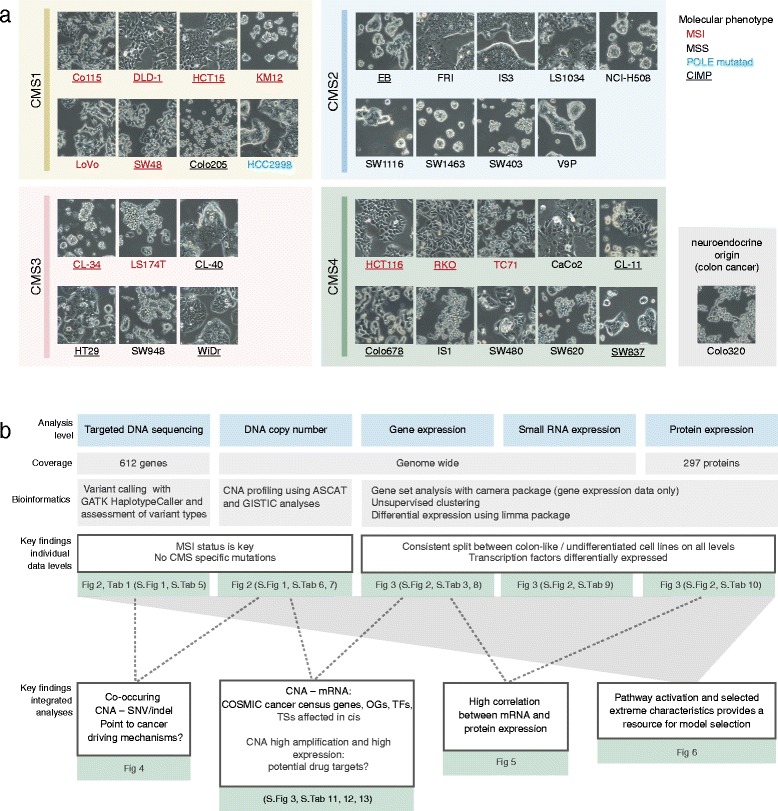
Overview of the 34 CRC cell lines analyzed and key findings. **a** The cell lines are grouped according to the gene expression-based CMSs (except Colo320, which has a neuroendocrine origin), and MSI, *POLE* and CIMP status are indicated. In general, the morphologic appearance of cell lines in CMS1 and CMS4 (for example LoVo and RKO) was mesenchymal, whereas cell lines in CMS2 and CMS3 (for example IS3 and WiDr) appeared more epithelial-like. **b** The cell lines were analyzed on the DNA, RNA and protein levels as indicated (*blue background*). Bioinformatic analyses (*grey*) were performed both on individual data levels and by integration of two or more data levels. Key findings (*white*) and references to figures and tables with detailed results are given (*green*). CIMP: CpG island methylator phenotype, CMS: consensus molecular subtypes, CNA: copy number aberrations, MSI/MSS: microsatellite instable/stable, OG: oncogene, TF: transcription factor, TS: tumor suppressor, SNV: single nucleotide variant

Thirty-four CRC cell lines purchased from cell line repositories or kindly provided by collaborators (Additional file 1: Table S1), were subjected to DNA, RNA and protein analyses (Fig. 1 a and b). Cell lines were cultured as previously described [12] and harvested at approximately 80–90% confluency. Genomic DNA was extracted either by a standard phenol/chloroform procedure or a magnetic beads protocol (Maxwell 16 DNA purification kit, Promega, Madison, WI, U.S.A.). Cell line authenticity was verified by DNA profiling based on 15 short tandem repeat (STR) loci, using the AmpFLSTR Identifiler PCR Amplification Kit (Thermo Fisher, Waltham, MA) and matched to the profiles from supplier (Additional file 1: Table S1) where available. MSI and CIMP status was determined according to previously described procedures [12]. For CL-40 MSI status was additionally assessed using the MSI Analysis System, version 1.2 (Promega, Fitchburg, WI, USA). Total RNA was extracted using the Qiagen AllPrep DNA/RNA/miRNA Universal kit (Qiagen, Hilden, Germany) and quality controlled by the Agilent RNA 6000 nano kit for Agilent 2100 Bioanalyzer (Agilent, Santa Clara, CA, U.S.A.). All RIN values were above 9. Protein lysates were produced from cell pellets at the MD Anderson Cancer Centre RPPA Core Facility.

For all cell lines, DNA copy number, mRNA, miRNA and protein expression profiles were generated. Targeted DNA sequencing was performed for 27 cancer cell lines in addition to Sanger sequencing of selected genes in 31 cell lines. For frequency counts and statistical tests we excluded the neuroendocrine Colo320 and kept only one cell line derived from the same patient, thus excluding IS3, SW620, DLD-1 and WiDr.

### Targeted deep sequencing

Sequencing libraries for the “kinome” and selected cancer-relevant genes (totally *n* = 612 genes; Additional file 1: Table S2) was generated using the Agilent SureSelect Human Kinome V1 kit (Agilent), and 2 × 101 basepair paired-end sequencing was performed on the Illumina HiSeq 2500 system (Illumina, San Diego, CA, U.S.A.) at the Oslo University Hospital Genomics Core Facility to an average sequencing depth of 161X (range 105-289X). Sequencing reads were aligned to the reference genome GRCh37 (hg19) with the Burrows-Wheeler Aligner (v0.6.21) [16], converted from sequence alignment map (SAM) files to the binary alignment map (BAM) format by Picard, version 1.61 [17], and sorted and indexed using SAMtools (v0.1.18) [18]. Duplicate reads were removed by Picard, and the Genome Analysis Toolkit (GATK, v2.7–4) [19, 20] was used for local realignment around indels. Variant calling of both SNVs and indels was done using the HaplotypeCaller tool from GATK, and candidate variants were annotated using ANNOVAR (build 2013–02-21) [21]. Only variants with minimum 10 alternative reads were included in further analysis. As sequencing analyses on cell lines do not enable filtering of germ line variants, candidate variants present in dbSNP version 138 [22] and not marked as clinically associated in this version of dbSNP, were discarded, and the final cell line “mutations” are referred to as variants throughout the paper. The following variants were defined as non-synonymous: non-synonymous SNVs, stopgain SNVs, stoploss SNVs and frameshift indels.

Sanger sequencing was performed for the whole coding sequences of *PTEN* and *TP53* and for mutation hotspots in *KRAS* codons G12, G13, Q61, K117 and A146, *BRAF* V600 and *PIK3CA* E542, E545, E546, H1025 and H1047 for seven of the cell lines. The mutation statuses for most of the codons above for the remaining 24 cell lines are described previously [12], except for *KRAS* codons K117, A146 and *PIK3CA* codon and H1025, which are included in the current work. Colo205, HCC2998 and KM12 were not assessed by Sanger sequencing.

### High resolution DNA copy number profiles

DNA copy number data was generated using Affymetrix Genome-Wide Human SNP 6.0 microarrays (Affymetrix Inc., Santa Clara, CA). One μg of DNA in low-EDTA TE-buffer was prepared according to the Affymetrix SNP 6.0 Cytogenetics Copy Number Assay User Guide and hybridized to Affymetrix Genome-Wide SNP 6.0 microarrays according to the Affymetrix Genome-Wide Human SNP Nsp/Sty User Guide. Resulting raw data were within recommended QC thresholds (CQC > 0.4; MAPD < 0.35). Signal extraction and pre-processing of raw data was performed as previously described [23], using the PennCNV protocol modified for Affymetrix genotyping arrays with Affymetrix Power Tools version 1.15.0 [24, 25] with HapMap samples as reference [26]. Single-sample segmentation of normalized and GC corrected data was done with the R package copynumber (version 1.14.0) [27]. The user defined penalty parameter was set to 100. PCF value thresholds were set to ≥0.15 (gain) and ≤ −0.15 (loss). To enable comparison of samples with different breakpoints, the smallest regions of overlap (SROs) were determined. Each SRO originated from a true larger segment and the copy number value of the originating segment was kept. Copy number estimates per gene were retrieved by mapping chromosomal segments from each sample to the R implemented transcript database TxDb.Hsapiens.UCSC.hg19.knownGene (v3.2.2), utilizing the findOverlaps function from the GenomicRanges R package (v1.22.4).

The percentage of the genome affected by copy number aberrations (CNAs) was defined as the percentage of bases with aberrant copy number out of the total number of bases with a copy number value available.

To detect potential CNA targets, The GISTIC algorithm v2.0.22 [28] was run with default parameters with the following exceptions: the threshold for broad events was set to 70% of the chromosome arm length; the maximum number of segments in a sample was set to 2000; the confidence level was set to 99%; parameters for gene-level and broad-level analysis was set ON.

### Gene expression analysis

Microarray gene expression analyses were performed using Affymetrix HTA 2.0 Transcriptome Arrays (Affymetrix Inc., Santa Clara, CA, U.S.A.), according to the manufacturer’s instructions. The data was normalized and summarized at the gene level using the Guanine Cytosine Count Correction and Signal Space Transformation algorithms with Robust Multi-array Average (SST-RMA) implemented in the Affymetrix Expression Console Software (v1.4.1, HTA-2_0.r3 library files). The HTA-2_0.na35.2.hg19.transcript.csv annotation file identified 67,528 annotated genes (transcript clusters). The data was filtered to exclude non-coding RNA probes, and genes annotated by multiple probesets were filtered to retain one probeset per gene by prioritizing annotation databases: RefSeq, ENSEMBL, other databases. The filtered dataset contained data for 18,740 probesets.

Principal component analysis (PCA) was performed including only the 1000 genes with the largest cross-sample variation. PC1 had a bimodal density distribution and samples with PC1 score larger than the between-peaks minima were defined as “high”. Gene set tests were performed using camera [29, 30]. Single sample Gene Set Enrichment Analysis (ssGSEA) was performed using GSVA [31]. Seventy gene sets were assembled to enrich for pathways likely to be informative on CRC biology based on Guinney et al. [8] (Additional file 1: Table S3). Differential expression analysis was performed using the R package limma [30].

The cell lines have been classified according to CMS based on the nearest predicted subtype, using an adapted classifier independent of gene expression signaling from the tumor microenvironment (Sveen et al., submitted).

The gene expression data has been submitted to the NCBI’s Gene Expression Omnibus with accession number GSE97023.

### Small RNA sequencing

Small RNA sequencing libraries were prepared using a recently published low-bias protocol [32] and resulting libraries subjected to sequencing on an Illumina HiSeq 2500 (rapid mode). The 50 bp single-end reads were de-multiplexed and converted to FASTQ files by Casava (v1.8.2). Reads were adapter trimmed, quality filtered and collapsed by FASTX Toolkit (v0.0.14). We discarded reads that met any of the following criteria; less than 7 bases matching adapter sequence, shorter than 18 bases after adapter clipping, and/or phred score below 27 for more than 8% of the bases. The eight randomized N-bases were removed prior to alignment. Processed reads were aligned against a custom reference of miRBase hairpins (v21) using bowtie (v1.1.1), allowing no mismatches. The reads were summarized over each mature miRNA, requiring at least 18 nt overlap using R packages GenomicRanges, rtracklayer and ShortRead. Differential expression analysis of miRNA data was performed with R package limma using voom with cyclic loess normalization [30, 33].

### High-throughput protein expression analysis

Reverse Phase Protein lysate Array (RPPA) analysis with 297 antibodies targeting 235/62 proteins/phospho-proteins (Additional file 1: Table S4) was performed at MD Anderson Cancer Centre RPPA Core Facility, including pre-processing of the protein data. Median centered normalized log_2_ values describing the relative protein abundance in each sample were used for downstream analyses. Differential expression analysis of RPPA data was performed with R package limma [30].

### Integration of DNA copy number and gene expression data

We explored the influence of *in-cis* copy number aberrations on gene expression by testing for differences in gene expression among CNA groups. For this analysis, a stricter PCF value threshold of ≥0.3 or ≤ − 0.3 was used to define gain and loss. Genes spanning several segments (differing in PCF value along the gene) were handled as follows: Genes consistently gained or lost (different PCF value but belonging to the same category) were assigned to the correct category accordingly. Genes that differed in copy number category (e.g. loss in one part, gain or neutral in remaining part) was assigned the median PCF value. Only genes represented in all samples were included. The mRNA expression was defined to be associated with copy number *in cis* if Wilcoxon testing determined (i) the mRNA expression to be significantly different in samples with gain versus samples with neutral copy number or loss, or (ii) the mRNA expression was significantly different in samples with loss versus samples with neutral copy number or gain. We corrected for multiple testing by false discovery rate (FDR) using the p.adjust function in the R stat package.

To limit false positives, genes within the lower quartile of mean gene expression were excluded and only genes with IQR > 0.7 were retained, *n* = 5120 genes for *in cis* analyses. Only unique MSS cell lines (*n* = 18) and genes with aberrant copy number > 2 cell lines were investigated (gain: 1148 genes; loss: 1047 genes). GO enrichment analysis of significant *in* cis genes was done with the PANTHER overrepresentation test with the GO consortium online tool [34]. Significant genes from *in cis* analyses were investigated for overlaps with the MSigDB version 5.2 [35, 36].

Associations between CNA and gene expression were additionally assessed by gene-wise Spearman correlations of copy number- and expression values across samples, and genes with correlations above 0.7 were considered to show an association.

To identify potential CNA drivers, we looked for outliers in CNA estimates corresponding to high or low *in-cis* expression in unique CIN cell lines. We applied a cutoff of 4 times gain/loss threshold (0.15/−0.15) to nominate potentially high amplitude CNAs, and gene expression values outside 3 times the standard deviation from sample mean expression across all genes were considered outliers and hence interesting. We looked for concurrent CNAs and gene expression events by retrieving genes for which the minimum/maximum CNA value and gene expression value belonged to the same cell line.

## Results

A panel of 34 CRC cell lines was analyzed at the DNA, RNA, and protein level (Fig. 1a; Additional file 1: Table S1). Results are presented in figures and tables for each individual data level and integration analysis, as summarized in Fig. 1b. The panel comprised 11 MSI and 22 MSS cell lines, in addition to the MSS *POLE* mutated HCC2998 [37]. The cell lines have previously been shown to recapitulate the biological properties of the four CMSs (Sveen et al., submitted). Out of the 34 cell lines, 8 were classified as CMS1-“immune”, 9 as CMS2-“canonical”, 6 as CMS3-“metabolic”, and 10 as CMS4-“mesenchymal”. Colo320 is derived from a neuroendocrine tumor and has a distinct gene expression profile [38].

### DNA sequence aberrations reflect hypermutator phenotypes

**Fig. 2:**
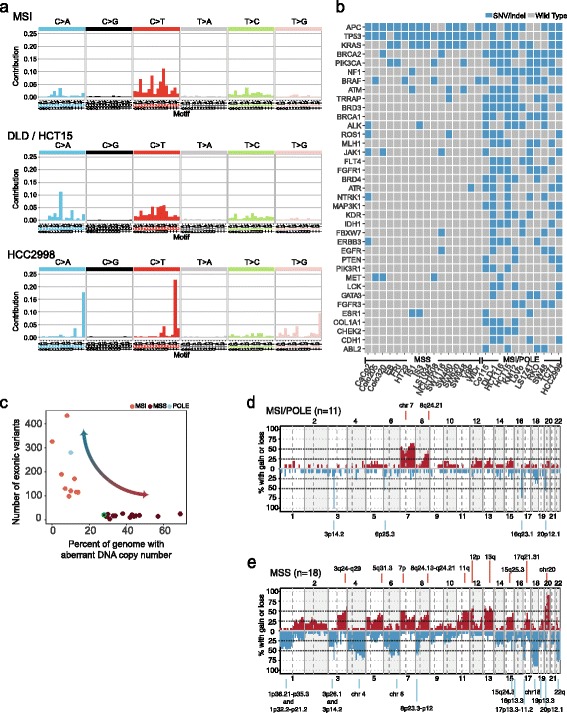
DNA aberrations reflect the type of genomic instability. **a** We investigated the frequencies (*vertical axes*) of SNVs in each of six categories (indicated in the top panels) grouped according to sequence motif (flanking nucleotides are indicated on the horizontal axes). MSI cell lines (*n* = 8, excluding DLD1 and HCT15) and the *POLE* mutated cell line HCC2998 displayed different mutation signatures associated with the respective types of hypermutation. The MSI cell lines DLD-1 and HCT15 had a distinct mutation signature with a combination of deficient mismatch repair and *POLD1* mutation. **b** Overview of detected SNVs/indels in 37 genes included in the Cosmic Cancer Gene Census and that were mutated in at least four MSI cell lines or one MSS cell line among the 27 cell lines analyzed by targeted deep sequencing. Most genes showed clear mutation frequency differences between MSS and MSI/*POLE* mutated cell lines. **c** There was an inverse relationship between the CNA load (horizontal axis; percent of basepairs with aberrant copy number) and the SNV/indel load (*vertical axis*) in the cell lines, reflecting their molecular subtype, as indicated. The neuroendocrine cell line Colo320 (*green circle*) grouped along with the MSS cell lines, and had few SNVs/indels and a moderate number of CNAs, including gain of 8q and 13q. **d** MSI/*POLE* mutated cell lines had a lower frequency of CNAs (*vertical axis*) along the genome than **e** MSS cell lines. In each plot, chromosomes are indicated on the horizontal axes and separated by vertical lines (whole and dashed lines for chromosomes and chromosome arms, respectively). Frequent aberrations are highlighted, including gains on 7p, 7q, 8q, 12p, 13q, 20q and losses on 4p, 4q, 17p, 18q and 22q, which are chromosome arms known to be frequently affected by CNAs in primary CRCs. CNA: copy number aberration, MSI/MSS: microsatellite instable/stable, POLE: *POLE* mutated, SNV: single nucleotide variant

Cell lines with a hypermutator phenotype associated with MSI or *POLE* mutation had a median of 126 (range 98–327) non-synonymous variants (SNVs or indels) in the 612 sequenced genes, significantly more than the 18 (range 4–26) found in MSS cell lines (*p* = 9∙10^−5^, Wilcoxon rank-sum test). This corresponds to 82 (range 63–212) and 12 (range 3–17) non-synonymous variants per million coding basepairs sequenced, respectively. For reference, The Cancer Genome Atlas reported approximately 1–300 somatic mutations per million basepairs for primary CRCs [39]. MSI cell lines had a high proportion of C > T variants, especially in an NpCpG sequence context, consistent with a mismatch repair deficiency mutation signature commonly found in MSI cancers (Signature 6; Fig. 2a) [40]. The MSI cell lines DLD-1 and HCT15 (derived from the same patient) had the highest variant loads, with 592 and 442 SNVs respectively (Additional file 2: Fig. S1a). In addition to the large proportion of MSI-associated C > T variants, these cell lines had a larger contribution of C > A variants in a CpCpT sequence context compared to other MSIs, recently reported to be caused by a *POLD1* R689W mutation (Fig. 2a) [41]. Consistently, DLD-1 and HCT15 also had a substantially lower number of indels relative to SNVs than other MSI cell lines (Additional file 2: Fig. S1a). The MSS cell line HCC2998, which has a *POLE* P286R substitution [37], had the third highest variant load with 281 non-synonymous variants. This cell line had few indels and a high proportion of C > A variants in a TpCpT context, C > T variants in a TpCpG and T > G variants in a TpTpT context, which are associated with the *POLE* hypermutator phenotype and mutation Signature 10 [40].

The genes that were most frequently affected by SNVs/indels and also listed in the COSMIC Cancer Gene Census are summarized in Fig. 2b. Selected variants in CRC critical genes, analyzed by Sanger and/or targeted sequencing, are presented in Table 1. None of the detected common variants were restricted to one CMS group, and variant frequencies rather reflected the MSI status of the cell lines. A complete list of the detected exonic non-synonymous SNVs and indels is found in Additional file 1: Table S5.

**Table 1:** Mutation status in CRC critical genes. Cell lines were examined by Sanger sequencing, targeted sequencing or by both methods

	TP53	KRAS	BRAF	PIK3CA	PTEN	MSI	CIMP
CaCo2	p.E204X	wt	wt	wt	wt	MSS	CIMP-
CL-11^a^	p.S215N	p.V14I; p.Q61H	wt	wt	wt	MSS	CIMP+
CL-34^a^	p.S127P; p.K382fs	wt	p.V600E	wt	wt	MSI	CIMP+
CL-40^a^	p.R248Q	p.G12D	wt	wt	wt	MSS	CIMP+
Co115	wt	wt	p.V600E	wt	p.E157fs; p.R233X	MSI	CIMP+
Colo205^b^	p.Y107fs; p.Y103fs	wt	p.V600E	wt	wt	MSS	CIMP+
Colo320	p.R248W	wt	wt	wt	wt	MSS	CIMP-
Colo678^a^	wt	p.G12D	wt	wt	wt	MSS	CIMP+
DLD-1	p.S241F	p.G13D	wt	p.E545K; p.D549N	wt	MSI	CIMP+
EB	wt	p.G12D	wt	p.E545K	wt	MSS	CIMP+
FRI	p.C277F	p.G13D	wt	p.E545K	wt	MSS	CIMP-
HCC2998^b^	p.R213X	p.A146T	wt	wt	p.Y46C; p.R130Q; p.F341V	MSS	CIMP-
HCT116	wt	p.G13D	wt	p.H1047R	wt	MSI	CIMP+
HCT15	p.S241F	p.G13D	wt	p.E545K; p.D549N	wt	MSI	CIMP+
HT29	p.R273H	wt	p.V600E; p.T119S^c^	wt	wt	MSS	CIMP+
IS1	p.Y163H	p.G12D	wt	wt	wt	MSS	CIMP-
IS3	p.Y163H	p.G12D	wt	wt	wt	MSS	CIMP-
KM12^b^	p.P72fs; p.H179R	wt	p.P403fs	wt	p.G129X; p.K267fs	MSI	CIMP+
LoVo	wt	p.G13D; p.V14A	wt	wt	wt	MSI	CIMP-
LS1034	p.G245S	p.A146T	wt	wt	wt	MSS	CIMP-
LS174T	wt	p.G12D	p.D211G^c^	p.H1047R	wt	MSI	CIMP-
NCI-H508	p.R273H	wt	p.G596R	p.E545K	wt	MSS	CIMP-
RKO	wt	wt	p.V600E	p.H1047R	wt	MSI	CIMP+
SW1116	p.A159D	p.G12A	wt	wt	wt	MSS	CIMP-
SW1463^a^	p.R248Q	p.G12C	wt	wt	wt	MSS	CIMP-
SW403^a^	p.E51X	p.G12V	wt	wt	wt	MSS	CIMP-
SW48	wt	wt	p.R347X^c^	p.G914R^c^	wt	MSI	CIMP+
SW480	p.R273H; p.P309S	p.G12V	wt	wt	wt	MSS	CIMP-
SW620	p.R273H; p.P309S	p.G12V	wt	wt	wt	MSS	CIMP-
SW837^a^	p.R248W	p.G12C	wt	wt	wt	MSS	CIMP+
SW948	p.G117fs	p.Q61L	wt	p.E542K	wt	MSS	CIMP-
TC71	p.C176Y; p.R213X	p.G12D	wt	p.R88Q^c^	p.R233X	MSI	CIMP-
V9P	p.G245D	wt	wt	wt	wt	MSS	CIMP-
WiDr	p.R273H	wt	p.V600E; p.T119S^c^	wt	wt	MSS	CIMP+

All variants found with targeted sequencing and not observed in Sanger sequencing data were found in regions outside codons targeted by Sanger. HT29/WiDr had two variants each in *BRAF*, where one was verified by Sanger, and the other was outside of codons assessed. Only non-synonymous mutations were reported from targeted sequencing data

^**a**^Mutation data were available from Sanger sequencing only

^**b**^Mutation data were available from targeted sequencing only

^**c**^Mutations found with targeted sequencing only, but outside of regions assessed by Sanger sequencing

### DNA copy number aberrations reflect the CIN phenotype

We confirmed an inverse relationship between the number of SNVs/indels and DNA copy number aberrations (CNAs, % genome affected), reflecting the type of genomic instability (Spearman’s rho = −0.74, *p* = 1∙10^−5^; Fig. 2c). MSI/*POLE* mutated cell lines had significantly less CNAs (range 0–14%, median 9%) compared to MSS cell lines (range 12–69%, median 40%; Wilcoxon rank-sum test, *p* < 2.2∙10^−16^, Additional file 2: Figure S1b; Additional file 1: Table S1). CL-40, which is previously reported to have MSI [14], was here found to be MSS, but the number of CNAs was low and the cell line may thus represent a non-CIN non-MSI phenotype (12% genome affected by CNAs). CMS1 cell lines had fewer CNAs (range 0–45%, median 10%) compared to CMS2/3/4 (range 7–69%, median 32%; Wilcoxon rank-sum test, *p* = 0.01), reflecting the high prevalence of MSI in the CMS1 subtype, and CMS2 cell lines had more CNAs compared to CMS1/3/4, although not statistically significant (CMS2 range 27–59, median 33%; CMS1/3/4 range 0–69, median 13%; *p* = 0.06).

Although cell lines with MSI or *POLE* mutation (*n* = 11) harbored few DNA copy-number aberrations, two broad gains and four focal losses were observed with frequencies higher than 40% (Fig. 2d). In contrast, MSS cell lines (*n* = 18) had 24 separate regions affected in more than 40% of the cell lines (Fig. 2e). CNAs detected in MSI cell lines were not exclusive for this subtype, although the focal losses were less frequent in MSS cell lines.

Potential target genes of CNAs were identified in 7 and 23 focal areas of gain and loss respectively (GISTIC analysis, q-value < 0.25; Additional file 1: Table S6), including *KLF5* (gain 13q), *GPHN* (loss 14q) and *SMAD4* (loss 18q), as well as genes located in known fragile genomic areas, like *FHIT* (3p), *WWOX* (16q) and *MACROD2* (20p).

No copy number changes were restricted to one CMS group (MSS only; Additional file 2: Figure S1c). Some CNAs were more recurrent in undifferentiated MSS cell lines (*n* = 6, mainly CMS1 and CMS4 cell lines) compared to the colon-like MSS cell lines (*n* = 12, mainly CMS2 and CMS3) and vice versa. This included higher frequency of chromosomes 8 and 13 gain and loss of focal regions on 3p, 4q, 14q, 17p, 20p and 22q in colon-like cell lines and gain of 5q and 22q in undifferentiated cell lines (Additional file 2: Figure S1d). A genome wide overview of gene copy number status for all cell lines is presented (Additional file 1: Table S7).

### mRNA, miRNA and protein expression profiles are distinct between undifferentiated and “colon-like” cell lines

**Fig. 3:**
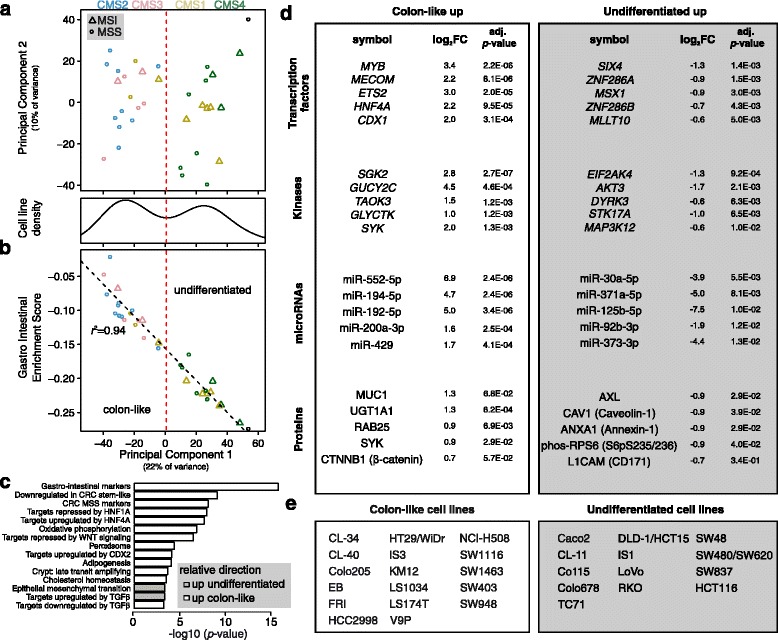
Gene expression based classification of CRC cell lines revealed a separation between colon-like and undifferentiated cell lines associated with the consensus molecular subtypes (CMS). **a** PCA of cell line mRNA expression data (plotted as sample-wise PC1 versus PC2) showed that the cell lines had a bimodal density distribution along PC1 (*bottom plot*), indicating two distinct subgroups largely separating CMS2/3 from CMS1/4. Each point represents one cell line, and is colored according to the CMS class and with point type indicating MSI-status. Dashed vertical line (*red*) indicates the least frequent value between the two density modes of PC1, and was used as a threshold to separate the cell lines into the two subgroups. **b** PC1 (*horizontal axis*) was strongly correlated with the sample-wise enrichment score for a set of gastro-intestinal tissue enhanced genes (*vertical axis*), and cell lines with high enrichment scores, left of the red dashed line, were termed “colon-like” and the remaining “undifferentiated”. **c** Gene set enrichment analyses comparing colon-like and undifferentiated cell lines showed that colon-like cell lines had higher expression of genes upregulated by HNF4A and lower expression of genes related to colorectal cancer stemness. Undifferentiated cell lines had higher expression of genes related to epithelial to mesenchymal transition and genes upregulated by TGFβ. The plot includes the top 15 gene sets tested (ranked by *p*-value) and the -log_10_ *p*-value is plotted on the horizontal axis. **d** Top 5 differentially expressed transcription factors and kinases (mRNA level), miRNAs and proteins between colon-like and undifferentiated cell lines. mRNAs and miRNAs are ranked by *p*-value while proteins are ranked by absolute log_2_ fold-change. The log_2_ fold-changes (log_2_FC) between the sample groups are indicated. **e** Classification of the individual cell lines according to the colon-like and undifferentiated subgroups. CRC: colorectal cancer, CMS: consensus molecular subtypes, log_2_FC: log_2_ fold-change, MSI/MSS: microsatellite instable/stable, PCA: principal component analysis

Unsupervised PCA of mRNA expression data showed that the cell lines formed two distinct clusters, as highlighted by the bimodal density distribution of samples along the first principal component (PC1, Fig. 3a). A similar pattern was apparent also in the miRNA and protein expression datasets (Additional file 3: Figure S2a). To explore the biological basis for this separation, we correlated PC1 from mRNA expression data to single-sample gene set enrichment analysis (ssGSEA) scores for 70 pre-selected CMS and CRC relevant gene sets (Additional file 1: Table S3). The top hit was a gastro-intestinal tissue enhanced gene set, derived from The Human Protein Atlas [42], with the ssGSEA score explaining more than 90% of the variance along PC1 (*r* ^2^ *=* 0.92, *p <* 2∙10^−16^, Pearson’s correlation, Fig. 3b). We used the PC1 density to classify the cell lines with low PC1/high gastro-intestinal ssGSEA score as *colon-like* and the remaining as *undifferentiated* (18 and 15 cell lines, respectively)*.* This grouping was significantly associated with the CMS groups (CMS2/3 versus CMS1/4), but less so with MSI-status (*p* = 2∙10^−6^ and *p* = 0.06, respectively, Fisher’s exact test). The finding was corroborated by morphological appearances; for example the undifferentiated cell lines LoVo and RKO appeared more mesenchymal, while colon-like IS3 and WiDr formed large epithelial-like sheets in culture (Fig. 1a). To further characterize the differences between colon-like and undifferentiated cell lines, we performed gene set analysis [29]. Out of 70 gene sets, 17 showed a significant relative difference (FDR corrected *p* < 0.05, Fig. 3c and Additional file 1: Table S3). In addition to the gastro-intestinal markers, colon-like cell lines were characterized by relative upregulation of genes positively regulated by the HNF4A and CDX2 transcription factors and genes repressed by HNF1A and WNT signaling (Fig. 3c). Conversely, undifferentiated cell lines had higher epithelial-to-mesenchymal transition (EMT) signature score and increased expression of TGFβ induced genes (Fig. 3c).

To pinpoint important factors maintaining the distinction between colon-like and undifferentiated cell lines, we performed differential mRNA, miRNA and protein expression analysis (Additional file 3: Figure S2b, Additional file 1: Tables S8, S9, S10). At the mRNA level, *CEACAM5,* which encodes a carcinoembryonic antigen (CEA) protein used as a blood-based biomarker for monitoring CRC patients, was more than 100-fold higher in colon-like cell lines. In undifferentiated cell lines, *TGFB1* and *TGFB2* were 3- and 7-fold higher, respectively. The five transcription factors with the most significant upregulation in colon-like cell lines were *MYB, MECOM, ETS2, HNF4A* and *CDX1* (Fig. 3d), consistent with high expression of these genes in human gastro-intestinal tissues [42]. The most significantly upregulated transcription factors in undifferentiated cell lines were *SIX4*, *ZNF286A*, *MSX1*, *ZNF286B* and *MLLT10*.

Differential miRNA expression analysis showed upregulation of the miRNAs encoded in the mir-194 ~ 192 and mir-200b ~ 429 clusters in colon-like compared to undifferentiated cell lines. MiRNAs in the mir-194 ~ 192 cluster are highly specific to colonic tissue [43] while miR-200 is critical in establishing and maintaining epithelial cell identity [44], corroborating the mRNA-based subgroup designations. Among proteins analyzed, AXL, CAV1, ANXA1, phosphorylated RPS6 and L1CAM (CD171) were highly upregulated in undifferentiated cell lines (Fig. 3d, Additional file 3: Figure S2b). For colon-like samples, MUC1, UGT1A, RAB25, SYK and β-catenin (CTNNB1) had the largest fold-change when compared to undifferentiated cell lines, but also E-cadherin (CDH1) and EGFR were significantly upregulated.

Summarized, CRC cell lines form two major biologically distinct expression subgroups at the mRNA, miRNA and protein level, which are distinguished by the expression of gastro-intestinal and epithelial differentiation markers.

### Integrated analysis identifies in vitro models for studies of targetable genes

To detect genes and pathways repeatedly affected by different aberrations in individual cell lines, we integrated data from different genomic levels, focusing on central CRC pathways and transcription factors.

#### Concurrent CNAs and SNVs/indels in cancer critical genes

**Fig. 4:**
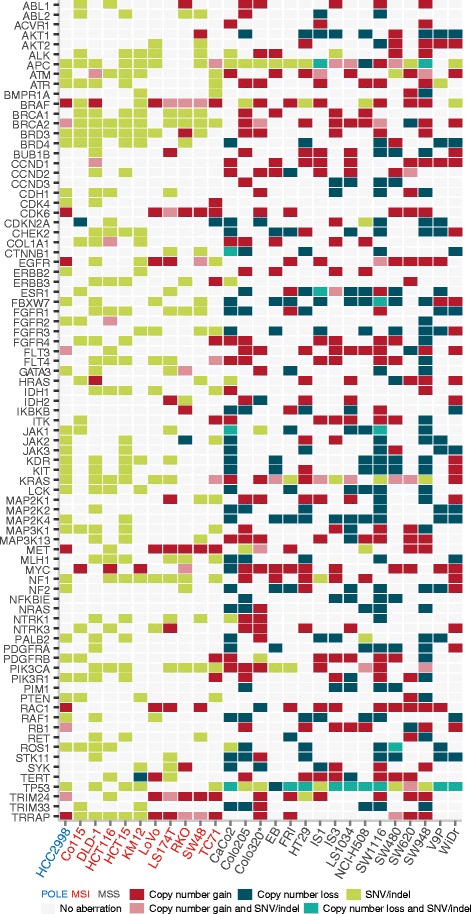
CNAs and SNVs/indels in cancer-critical genes. Among genes in the Cancer Gene Census (*n* = 83 genes included in the targeted sequencing panel, ranked vertically in alphabetical order), simultaneous mutations and CNAs in individual cell lines (grouped horizontally according to genomic phenotypes as indicated) were detected in CRC relevant oncogenes, including *KRAS* and *EGFR*, and tumor suppressor genes, including *TP53* and *APC*. The cell line Colo320, which has a neuroendocrine origin, is marked by an asterisk. CNA: copy number aberration, CRC: colorectal cancer, MSI/MSS: microsatellite instable/stable, POLE: *POLE* mutated, SNV: single nucleotide variant

In some cell lines, a simultaneous SNV/indel and CNA in the same gene was observed, including gains and SNVs in the oncogenes *KRAS* (*n* = 6 cell lines) and *EGFR* (*n* = 2). A complete overview of SNVs/indels, CNAs and the combination of these events in individual cell lines is shown in Fig. 4 (*n* = 83 genes in the Cancer Gene Census represented in the targeted sequencing data).

#### mRNA expression of oncogenes and transcription factors is associated with DNA copy number in cis

A total of 298 (26%) out of 1148 genes with copy number gain had a significant *in cis* association between copy number state and gene expression (analyzed by Wilcoxon rank-sum test) (Additional file 1: Table S11). Out of the 298 genes, 215 (72%) had strong correlations between copy number estimates and gene expression (Spearman correlation >0.7), 10 of which were found in the COSMIC Cancer Gene Census and 25 defined as transcription factors in the Molecular Signatures Database (MSigDB; highlighted in Additional file 1: Table S11). Similarly, 229 out of 1047 (22%) genes with copy number loss had an *in cis* association with gene expression (Additional file 1: Table S11). Out of the 229, 174 (76%) showed strong correlations between copy number estimates and gene expression (Spearman correlation >0.7), and 8 were found in the COSMIC Cancer Gene Census and 15 defined as transcription factors in MSigDB (highlighted in Additional file 1: Table S11). For gained *in cis* genes, the largest fold enrichment from gene ontology analysis was found for genes involved in nucleic acid metabolic process and cellular protein metabolic process (>1.5-fold enrichment). Biological processes enriched among lost *in cis* genes were mitotic cell cycle process, protein transport and intracellular transport (>2-fold enrichment; Additional file 1: Table S12).

Among the genes with significant *in cis* copy number and gene expression regulation, 56 were differentially expressed between colon-like and undifferentiated cell lines, including the transcription factors *ELF1* and *KLF5* and the lysosomal marker *LAMP1* (higher expressed in colon-like cell lines; FDR corrected *p* < 0.05; Additional file 1: Table S11).

#### Gene amplification and outlier expression

Genes with high-level copy number amplification and concurrent outlier gene expression may represent potential driver genes and drug targets. We identified 280 such genes across 18 unique MSS cell lines (Additional file 1: Table S13). Of these, 22 genes were classified as transcription factors in MSigDB and 15 genes were found in the COSMIC Cancer Gene Census, including *ERBB2* (Colo678), *MYC* (SW480), *PPFIBP1* (IS1), and *RAD21* (HT29) (Additional file 4: Figure S3a). The cell line V9P had high-level amplification with concurrent high expression of 68 genes, 29 of which were located on 22q, including *SMARCB1, BCR* and *MIF* (Additional file 1: Table S13). Protein expression data were available for ten genes, confirming high expression also at the protein level of the majority, including ERBB2, CCNE1 and MYC, suggesting that these copy number events are functionally important (Additional file 4: Figure S3b).

#### Consistent expression regulation at the gene and protein level

**Fig. 5:**
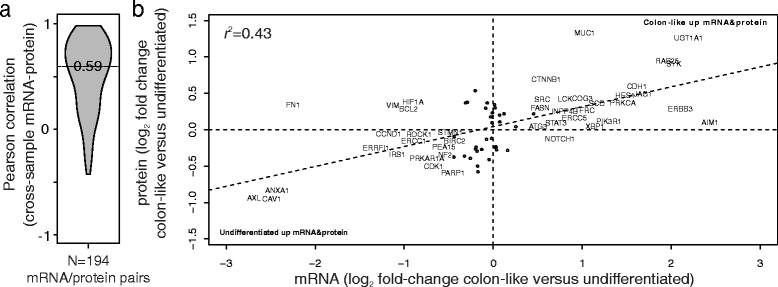
mRNA and protein expression levels are highly concordant among cell lines. **a** The density distribution (*horizontal axis*) of cross-cell line Pearson’s correlations (*vertical axis*) for expression of matched genes (microarray data) and proteins (Reverse Phase Protein Array data) (*n* = 194) shows an overall strong correlation. The horizontal line indicates the median correlation coefficient for all gene-protein pairs. **b** Differential expression analyses between colon-like and undifferentiated cell lines showed strong correspondence at the mRNA and protein level (plotted as the log_2_ fold-changes between the two groups of cell lines for matched protein on the vertical axis versus mRNA on the horizontal axis). The plot includes gene-protein pairs with adjusted *p*-value <0.1 from differential expression analysis in either mRNA or protein data. Gene-protein pairs with absolute log_2_ fold-change >0.5 (mRNA) between colon-like and undifferentiated cell lines are indicated by names and the rest by circles. Pearson correlation analysis (r^2^) indicated that 43% of the variance in the log_2_ fold-change at the protein level could be explained by mRNA-level log_2_ fold-change

To assess the correspondence between mRNA and protein-level expression (RPPA data) we calculated the Pearson’s correlations for each gene-protein pair among the cell lines (Fig. 5a). Excluding gene-protein pairs for which there was little variation among cell lines (lowest quartile in either dataset), the median correlation for all investigated pairs was 0.59 (IQR 0.26–0.78). AXL, CAV1, CDH1 (E-cadherin), EGFR and L1CAM had very strong correspondence, with correlation coefficients above 0.9. Similarly, mRNAs that were differentially expressed between colon-like and undifferentiated cell lines were generally also differentially expressed at the protein level (Fig. 5b).

### Multi-level data integration emphasizes molecular differences among cell lines relevant for functional studies

**Fig. 6:**
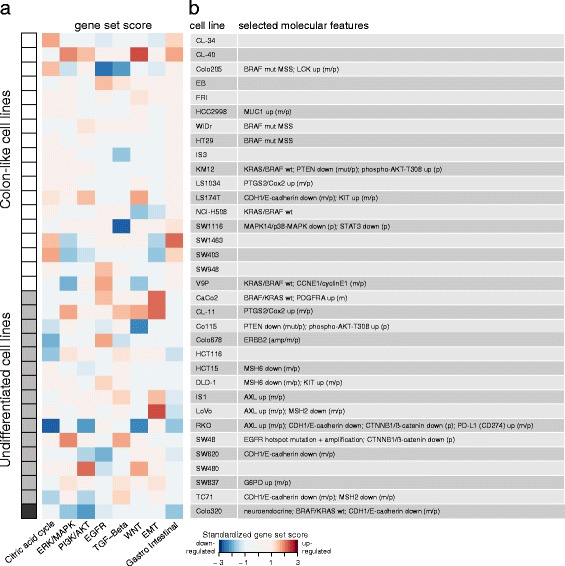
Characteristics of individual cell lines at multiple molecular levels. The cell lines are ranked alphabetically within the colon-like (*n* = 18; top) and undifferentiated (*n* = 15; bottom) subgroups. The neuroendocrine Colo320 is found below the undifferentiated cell lines (marked by a dark grey box). **a** The heatmap shows standardized single sample gene set expression enrichment scores for the eight selected pathways indicated at the bottom (indicates how many standard deviations the score is above or below the mean). Red indicates relative upregulation and blue indicates relative downregulation among cell lines. **b** The table indicates selected molecular events characteristic of each cell line. Amp: DNA amplification, mut: “mutation” (single nucleotide variant or insertion/deletion), m: mRNA level, p: protein level, wt: wild type

To facilitate selection of cell lines as appropriate research models, we performed gene expression enrichment analysis of eight gene sets representing important pathways or processes in CRC, including ERK/MAPK, PI3K/AKT, EGFR, TGFß and WNT signaling, in addition to signatures of epithelial to mesenchymal transition, citric acid cycle activation and the gastro-intestinal signature (Fig. 6a; Additional file 1: Table S14). The resulting heatmap indicates favorable systems for studying particular aspects. For example, SW1463 and CL40 are well-differentiated with low EMT-signature compared to CaCo2 and LoVo. Similarly, Colo205 and SW1116 have relatively low intrinsic TGFß activation in contrast to SW48 and CL-11. Finally, we assembled a list of outlier characteristics from the other data levels (Fig. 6b). Striking examples include high expression of the immune-suppressive protein PD-L1 in RKO, as well as mutation and downregulation of *PTEN* in KM12 and Co115, with concomitant hyper-phosphorylation of the AKT protein at residue T308.

## Discussion

CRC cell lines have previously been shown to recapitulate the mutational and transcriptional heterogeneity of primary tumors [7, 12, 14, 45]. Here we report an expanded overview of DNA, RNA and protein level characteristics of 34 CRC cell lines, analyzed in relation to genomic instability phenotypes and gene expression subgroups.

Consistent with known characteristics of the MSI and CIN phenotypes, we observed inverse levels of SNVs/indels and CNAs. All MSI cell lines had a mismatch repair deficiency-associated mutation signature, however, DLD-1 and HCT15 additionally had a high contribution from C > A variants in CpCpT trinucleotides and a low indel burden, a phenotype recently found to be the caused by the combination of MSI and a *POLD1* R689W mutation [41]. Although mutation analysis was restricted to a panel of 612 genes, the high mutation load in MSI/*POLE* mutated cell lines allowed for detection of expected mutation signatures. In the cell lines with a lower mutation load, broader sequencing coverage (whole exome or genome sequencing) would be more appropriate for accurate analyses of mutation processes. Unexpectedly, we find no evidence of MSI in CL-40, which has previously been reported as an MSI cell line [14]. This cell line also had a low number of CNAs, indicating that it may represent a non-MSI, non-CIN genomic phenotype.

Two distinct subgroups of CRC cell lines were evident at the mRNA, miRNA and protein expression levels. Based on gene set associations we termed these groups *colon-like* and *undifferentiated*. Colon-like cell lines were either CMS2 or CMS3, expressed higher levels of gastro-intestinal marker genes, including key transcription factors such as *HNF4A* and *MYB*. *HNF4A* has been nominated as a candidate driver for the 20q13.12 focal amplification peak suggesting a possible causal relationship between overexpression and expression subtype [39, 46]. Colon-like samples had in addition higher expression of mir-194 and mir-192, both highly enriched in colonic mucosa compared to other human tissues [43], independently supporting differentiation as a key distinction between the two cell line subgroups. Further, the miR-200 family, which represses the epithelial to mesenchymal transition program [44] was among the most abundant and most significantly upregulated miRNAs in the colon-like samples. Mir-200 promoter hypermethylation with concomitant downregulation was recently suggested to be a candidate marker for CMS4 tumors [47].

All CMS4 and most CMS1 models were classified as undifferentiated, consistent with primary tumors where CMS1 and CMS4 display a more stromal and undifferentiated signature [8]. As a group, undifferentiated cell lines showed relative upregulation of epithelial to mesenchymal transition signature and increased expression of *TGFβ* induced genes including *TGFβ1/2* cytokines. Recently it was shown that TGFβ signaling in cancer associated fibroblasts (CAFs) promotes tumor initiating capacity of CRC cells, and that CRC organoids with high TGFβ expression has a high metastatic potential [10]. As such, CMS1/CMS4 cancer cells may induce pro-metastatic behavior of CAFs through TGFβ1/2 paracrine signaling, illustrating how cancer cell-intrinsic expression may modulate the tumor microenvironment.

The notion that poorly differentiated tumors have inferior prognosis is not new [48–50] and the undifferentiated CMS4 is of particular clinical interest due to its association with poor prognosis. As such, the traits described here may be useful for further detailed studies of the biological background of this subtype. Also clinically relevant, undifferentiated cell lines expressed lower levels of CEA than colon-like cell lines, an observation which suggests that this biomarker may be less valuable in monitoring patients with CMS1 and CMS4 cancers.

Recurrently amplified chromosomal regions may harbor oncogenes that become overexpressed from the increase in gene-dosage [51]. About ¼ of genes had a good correlation between copy number state and gene expression level, some of which were also differentially expressed between colon-like and undifferentiated subtypes, such as the transcription factor *KLF5* (higher expressed in colon-like cell lines). Additionally, we identified high level amplifications with concurrent high gene expression in individual cell lines, including *ERBB2* in Colo678, also corroborated by high protein expression. The use of HER2-inhibitors together with the kinase inhibitor lapatinib was recently described as a treatment option in *HER2* amplified, *KRAS* wild-type metastatic CRC in a phase 2 trial [52]. We also observe that Colo678 have high *ADAM10* expression, suggested to be involved in acquired resistance to HER2-inhibition in breast cancer models. [53], and Colo678 may be used as a model system to elucidate resistance mechanisms for HER2 inhibition in CRC. V9P has few SNVs/indels, and drivers in this cell line are not well-explored. We found V9P to have concurrent amplification and high gene expression for more than 60 genes, including *CCNE1* (cyclin E1), which also had concurrent high protein expression levels. V9P represents a model for overexpression of cyclin E1, commonly observed in many cancers and which has been linked to chromosome instability [54, 55].

## Conclusion

By integration of DNA, RNA and protein data, we show that CRC cell lines represent consistent molecular subgroups defined by genomic instability phenotypes at the DNA level (sequence aberrations and CNAs) and differentiation at the expression level (mRNA, miRNA and protein). The data are made available per cell line in summary illustrations and detailed tables, and is a resource to select relevant models for further studies of cancer-cell intrinsic differences among CMS groups, functional biological mechanisms of CRC as well as pharmacogenomics.

## Additional files

Additional file 1: Tables S1-S14. Supplementary tables. (XLSX 4615 kb)

Additional file 2: Figure S1. DNA level aberrations. **a** SNVs and indel counts in 34 cell lines. MSI cell lines generally displayed numerous SNVs/indels, in contrast to MSS cell lines, although DLD-1/HCT15 were less typical with a lower indel burden compared to remaining MSIs. **b** The percentage of the genome with aberrant CNA reflects MSI status rather than CMS subtype. The figure includes 29 unique MSI/MSS cell lines. **c** CMS frequency of CNAs. Vertical axis indicates frequency, horizontal axes shows chromosomes 1–22, separated by vertical lines (whole lines separates chromosomes, dashed lines separates chromosome arms). The most common gains in CMS2 (5 or more out of 9 CMS2 MSI/MSS cell lines) were found on 3q, 8q, 13q, 17q, 20p and 20q, while regions of loss were frequent on 1p, 3p, 4q, 6p, 6q, 8p, 16p, 16q, 17p, 18 p, 18q, 20p and 22q. In CMS4 the most common gains (4 or more out of 7 CMS4 MSI/MSS cell lines) were found on 3q, 5p, 5q, 7p, 7q, 12p, 20p, 20q and 22q, while losses were frequent on 3p, 4p, 4q, 6q, 15q, 17p, 18q and 22q. The plots for CMS2 and CMS4 are placed together for easier visual comparison. A frequency plot for CMS3 was included, but the low sample number limits interpretations of frequent alterations in this group. **d** Differential frequencies of CNAs in undifferentiated versus colon-like cell lines. The vertical axis indicates the frequency difference between undifferentiated – colon-like cell lines (i.e. the frequency in undifferentiated cell lines minus the frequency of aberration in colon-like cell lines). The horizontal axis indicates chromosomes 1–22 (chromosomes separated by whole lines, chromosome arms separated by dashed lines). Yellow areas represent regions with higher frequencies of CNAs in colon-like cell lines, purple areas represent regions with higher frequencies of CNAs in undifferentiated cell lines. CMS: consensus molecular subtype, CNA: copy number aberration, MSI: microsatellite instable, MSS: microsatellite stable, SNV: single nucleotide variant. (PDF 830 kb)

Additional file 3: Figure S2. Expression differences between colon-like and undifferentiated cell lines. **a** PCA plots show the spontaneous split between the two subgroups in all three datasets (mRNA, miRNA and protein). **b** Volcano plots show differentially expressed genes in undifferentiated (*blue*) versus colon-like (*yellow*) cell lines on the mRNA, miRNA and protein levels. Horizontal dashed lines mark the highest *p*-value that produces an adjusted *p*-value of <0.01. Vertical dashed lines mark log_2_ fold-change (1 for mRNA/miRNA, 0.1 for protein). The top five differentially expressed mRNA/miRNA/proteins in terms of log_2_ fold-change within these thresholds are indicated by names, and the rest by filled circles. PCA: principal component analysis, PC1: principal component 1, PC2: principal component 2. (PDF 1095 kb)

Additional file 4: Figure S3. Outlier analysis reveals high level amplification events associated with marked mRNA expression changes, pointing to potential driver genes in individual cell lines. **a** A total of 280 genes were nominated, figure shows 15 nominated genes overlapping with the COSMIC Cancer Gene Census. Vertical axis shows gene expression values (log_2_ scale) and horizontal axis shows copy number aberration values (PCF values, log_2_ scale). The analysis pinpointed CNA values that were substantially higher in one or a few cell lines compared to remaining cell lines and hence 14 of the nominated genes were high amplification events, while one gene (SS18) was nominated on basis of all samples having loss except V9P. **b** Ten outlier genes had available associated protein data and for most genes the increase in gene expression was consistent on the protein level. Vertical axis shows relative protein expression (median centered normalized log_2_ values), horizontal axis shows gene expression values (log_2_ scale). (PDF 1233 kb)

## Abbreviations

CAFs: cancer associated fibroblasts
CEA: carcinoembryonic antigen
CIMP: CpG island methylator phenotype
CIN: chromosomal instability
CMS: consensus molecular subtype
CNA: copy number aberration
CRC: colorectal cancer
EMT: epithelial-to-mesenchymal transition
FDR: false discovery rate
Indel: insertion or deletion
miRNA: micro RNA
MSI: microsatellite instability
MSigDB: molecular signatures database
MSS: microsatellite stability
PCA: principal component analysis
RMA: robust multi-array average
RPPA: reverse phase protein lysate array
SNV: single nucleotide variant
SRO: smallest region of overlap
ssGSEA: single sample gene set enrichment analysis
SST: signal space transformation

## References

[1] Boland CR, Goel A (2010). Microsatellite instability in colorectal cancer. Gastroenterology.

[2] Pino MS, Chung DC (2010). The chromosomal instability pathway in colon cancer. Gastroenterology.

[3] Toyota M, Ahuja N, Ohe-Toyota M, Herman JG, Baylin SB, Issa JP (1999). CpG island methylator phenotype in colorectal cancer. Proc Natl Acad Sci U S A.

[4] Weisenberger DJ, Siegmund KD, Campan M, Young J, Long TI, Faasse MA (2006). CpG island methylator phenotype underlies sporadic microsatellite instability and is tightly associated with BRAF mutation in colorectal cancer. Nat Genet.

[5] Marisa L, de Reynies A, Duval A, Selves J, Gaub MP, Vescovo L (2013). Gene expression classification of colon cancer into molecular subtypes: characterization, validation, and prognostic value. PLoS Med.

[6] De Sousa F, Melo E, Wang X, Jansen M, Fessler E, Trinh A, de Rooij LPMH (2013). Poor-prognosis colon cancer is defined by a molecularly distinct subtype and develops from serrated precursor lesions. Nat Med.

[7] Sadanandam A, Lyssiotis CA, Homicsko K, Collisson EA, Gibb WJ, Wullschleger S (2013). A colorectal cancer classification system that associates cellular phenotype and responses to therapy. Nat Med.

[8] Guinney J, Dienstmann R, Wang X, de Reynies A, Schlicker A, Soneson C (2015). The consensus molecular subtypes of colorectal cancer. Nat Med.

[9] Isella C, Terrasi A, Bellomo SE, Petti C, Galatola G, Muratore A (2015). Stromal contribution to the colorectal cancer transcriptome. Nat Genet.

[10] Calon A, Lonardo E, Berenguer-Llergo A, Espinet E, Hernando-Momblona X, Iglesias M (2015). Stromal gene expression defines poor-prognosis subtypes in colorectal cancer. Nat Genet.

[11] Barretina J, Caponigro G, Stransky N, Venkatesan K, Margolin AA, Kim S (2012). The cancer cell line encyclopedia enables predictive modelling of anticancer drug sensitivity. Nature.

[12] Ahmed D, Eide PW, Eilertsen IA, Danielsen SA, Eknaes M, Hektoen M (2013). Epigenetic and genetic features of 24 colon cancer cell lines. Oncogene.

[13] Mouradov D, Sloggett C, Jorissen RN, Love CG, Li S, Burgess AW (2014). Colorectal cancer cell lines are representative models of the main molecular subtypes of primary cancer. Cancer Res.

[14] Medico E, Russo M, Picco G, Cancelliere C, Valtorta E, Corti G (2015). The molecular landscape of colorectal cancer cell lines unveils clinically actionable kinase targets. Nat Commun.

[15] Lind GE, Thorstensen L, Lovig T, Meling GI, Hamelin R, Rognum TO (2004). A CpG island hypermethylation profile of primary colorectal carcinomas and colon cancer cell lines. Mol Cancer.

[16] Li H, Durbin R (2009). Fast and accurate short read alignment with burrows-wheeler transform. Bioinformatics.

[17] Picard. http://broadinstitute.github.io/picard/. Accessed 5 Sept 2015.

[18] SAMtools. http://samtools.sourceforge.net/ Accessed 5 Sept 2015.

[19] DePristo MA, Banks E, Poplin R, Garimella KV, Maguire JR, Hartl C (2011). A framework for variation discovery and genotyping using next-generation DNA sequencing data. Nat Genet.

[20] McKenna A, Hanna M, Banks E, Sivachenko A, Cibulskis K, Kernytsky A (2010). The genome analysis Toolkit: a MapReduce framework for analyzing next-generation DNA sequencing data. Genome Res.

[21] Wang K, Li M, Hakonarson H (2010). ANNOVAR: functional annotation of genetic variants from high-throughput sequencing data. Nucleic Acids Res.

[22] Database of Single Nucleotide Polymorphisms (dbSNP). Bethesda (MD): National Center for Biotechnology Information, National Library of Medicine. (dbSNP Build ID: version 138). http://www.ncbi.nlm.nih.gov/SNP/ Accessed 15 Dec 2016.

[23] Sveen A, Loes IM, Alagaratnam S, Nilsen G, Holand M, Lingjaerde OC (2016). Intra-patient inter-metastatic genetic heterogeneity in colorectal cancer as a key determinant of survival after curative liver resection. PLoS Genet.

[24] PennCNV. http://penncnv.openbioinformatics.org/en/latest/user-guide/affy/ Accessed 15 Dec 2016.

[25] Wang K, Li M, Hadley D, Liu R, Glessner J, Grant SF (2007). PennCNV: an integrated hidden Markov model designed for high-resolution copy number variation detection in whole-genome SNP genotyping data. Genome Res.

[26] McCarroll SA, Kuruvilla FG, Korn JM, Cawley S, Nemesh J, Wysoker A (2008). Integrated detection and population-genetic analysis of SNPs and copy number variation. Nat Genet.

[27] Nilsen G, Liestol K, Van Loo P, Moen Vollan HK, Eide MB, Rueda OM (2012). Copynumber: efficient algorithms for single- and multi-track copy number segmentation. BMC Genomics.

[28] Mermel CH, Schumacher SE, Hill B, Meyerson ML, Beroukhim R, Getz G (2011). GISTIC2.0 facilitates sensitive and confident localization of the targets of focal somatic copy-number alteration in human cancers. Genome Biol.

[29] Wu D, Smyth GK (2012). Camera: a competitive gene set test accounting for inter-gene correlation. Nucleic Acids Res.

[30] Ritchie ME, Phipson B, Wu D, Hu Y, Law CW, Shi W (2015). Limma powers differential expression analyses for RNA-sequencing and microarray studies. Nucleic Acids Res.

[31] Hanzelmann S, Castelo R, Guinney J (2013). GSVA: gene set variation analysis for microarray and RNA-seq data. BMC Bioinf.

[32] Xu P, Billmeier M, Mohorianu I, Green D, Fraser William D, Dalmay T (2015). An improved protocol for small RNA library construction using high definition adapters. Methods in next generation sequencing.

[33] Law CW, Chen Y, Shi W, Smyth GK (2014). Voom: precision weights unlock linear model analysis tools for RNA-seq read counts. Genome Biol.

[34] Gene Ontology Consortium Enrichment Analysis. http://geneontology.org/page/go-enrichment-analysis Accessed 5 Dec 2016.

[35] Liberzon A, Birger C, Thorvaldsdóttir H, Ghandi M, Mesirov Jill P, Tamayo P (2015). The molecular signatures database Hallmark Gene set collection. Cell Systems.

[36] Molecular Signatures Database (MSigDB) version 5.2. http://software.broadinstitute.org/gsea/msigdb/gene_families.jsp Accessed 21 Oct 2016.

[37] Abaan OD, Polley EC, Davis SR, Zhu YJ, Bilke S, Walker RL (2013). The exomes of the NCI-60 panel: a genomic resource for cancer biology and systems pharmacology. Cancer Res.

[38] Quinn LA, Moore GE, Morgan RT, Woods LK (1979). Cell lines from human colon carcinoma with unusual cell products, double minutes, and homogeneously staining regions. Cancer Res.

[39] The Cancer Genome Atlas Research N (2012). Comprehensive molecular characterization of human colon and rectal cancer. Nature.

[40] Alexandrov LB, Nik-Zainal S, Wedge DC, Aparicio SA, Behjati S, Biankin AV (2013). Signatures of mutational processes in human cancer. Nature.

[41] Mertz TM, Baranovskiy AG, Wang J, Tahirov TH, Shcherbakova PV. Nucleotide selectivity defect and mutator phenotype conferred by a colon cancer-associated DNA polymerase delta mutation in human cells. Oncogene. 2017; doi:10.1038/onc.2017.1022. 10.1038/onc.2017.22 PMC5542868 28368425

[42] Uhlen M, Fagerberg L, Hallstrom BM, Lindskog C, Oksvold P, Mardinoglu A (2015). Proteomics. Tissue-based map of the human proteome. Science.

[43] Ludwig N, Leidinger P, Becker K, Backes C, Fehlmann T, Pallasch C, et al. Distribution of miRNA expression across human tissues. Nucleic Acids Res. 2016;44:3865–77. 10.1093/nar/gkw116 PMC4856985 26921406

[44] Park S-M, Gaur AB, Lengyel E, Peter ME (2008). The miR-200 family determines the epithelial phenotype of cancer cells by targeting the E-cadherin repressors ZEB1 and ZEB2. Genes Dev.

[45] Kleivi K, Teixeira MR, Eknæs M, Diep CB, Jakobsen KS, Hamelin R (2004). Genome signatures of colon carcinoma cell lines. Cancer Genet Cytogenet.

[46] Zhang B, Wang J, Wang X, Zhu J, Liu Q, Shi Z (2014). Proteogenomic characterization of human colon and rectal cancer. Nature.

[47] Fessler E, Jansen M, De Sousa F, Melo E, Zhao L, Prasetyanti PR, Rodermond H (2016). A multidimensional network approach reveals microRNAs as determinants of the mesenchymal colorectal cancer subtype. Oncogene.

[48] Benson AB, Schrag D, Somerfield MR, Cohen AM, Figueredo AT, Flynn PJ (2004). American Society of Clinical Oncology recommendations on adjuvant chemotherapy for stage II colon cancer. J Clin Oncol.

[49] Engstrom PF, Arnoletti JP, Benson AB, Chen YJ, Choti MA, Cooper HS (2009). NCCN clinical practice guidelines in Oncology: colon cancer. J Natl Compr Cancer Netw.

[50] Labianca R, Nordlinger B, Beretta GD, Brouquet A, Cervantes A (2010). Primary colon cancer: ESMO clinical practice guidelines for diagnosis, adjuvant treatment and follow-up. Ann Oncol.

[51] Albertson DG (2006). Gene amplification in cancer. Trends Genet.

[52] Sartore-Bianchi A, Trusolino L, Martino C, Bencardino K, Lonardi S, Bergamo F (2016). Dual-targeted therapy with trastuzumab and lapatinib in treatment-refractory, KRAS codon 12/13 wild-type, HER2-positive metastatic colorectal cancer (HERACLES): a proof-of-concept, multicentre, open-label, phase 2 trial. Lancet Oncol.

[53] Feldinger K, Generali D, Kramer-Marek G, Gijsen M, Ng TB, Wong JH (2014). ADAM10 mediates trastuzumab resistance and is correlated with survival in HER2 positive breast cancer. Oncotarget.

[54] Minella AC, Swanger J, Bryant E, Welcker M, Hwang H, Clurman BE (2002). p53 and p21 form an inducible barrier that protects cells against Cyclin E-cdk2 deregulation. Curr Biol.

[55] Spruck CH, Won K-A, Reed SI (1999). Deregulated cyclin E induces chromosome instability. Nature.

