# Supplementary material for: Multi-omics of 34 colorectal cancer cell lines - a resource for biomedical studies
Source: Mol Cancer. 2017 Jul 6;16:116. doi: 10.1186/s12943-017-0691-y (PMC5498998; doi:10.1186/s12943-017-0691-y)
Supplement: Supplementary file 2 — DNA level aberrations. a SNVs and indel counts in 34 cell lines. MSI cell lines generally displayed numerous SNVs/indels, in contrast to MSS cell lines, although DLD-1/HCT15 were less typical with a lower indel burden compared to remaining MSIs. b The percentage of the genome with aberrant CNA reflects MSI status rather than CMS subtype. The figure includes 29 unique MSI/MSS cell lines. c CMS frequency of CNAs. Vertical axis indicates frequency, horizontal axes shows chromosomes 1–22, separated by vertical lines (whole lines separates chromosomes, dashed lines separates chromosome arms). The most common gains in CMS2 (5 or more out of 9 CMS2 MSI/MSS cell lines) were found on 3q, 8q, 13q, 17q, 20p and 20q, while regions of loss were frequent on 1p, 3p, 4q, 6p, 6q, 8p, 16p, 16q, 17p, 18 p, 18q, 20p and 22q. In CMS4 the most common gains (4 or more out of 7 CMS4 MSI/MSS cell lines) were found on 3q, 5p, 5q, 7p, 7q, 12p, 20p, 20q and 22q, while losses were frequent on 3p, 4p, 4q, 6q, 15q, 17p, 18q and 22q. The plots for CMS2 and CMS4 are placed together for easier visual comparison. A frequency plot for CMS3 was included, but the low sample number limits interpretations of frequent alterations in this group. d Differential frequencies of CNAs in undifferentiated versus colon-like cell lines. The vertical axis indicates the frequency difference between undifferentiated – colon-like cell lines (i.e. the frequency in undifferentiated cell lines minus the frequency of aberration in colon-like cell lines). The horizontal axis indicates chromosomes 1–22 (chromosomes separated by whole lines, chromosome arms separated by dashed lines). Yellow areas represent regions with higher frequencies of CNAs in colon-like cell lines, purple areas represent regions with higher frequencies of CNAs in undifferentiated cell lines. CMS: consensus molecular subtype, CNA: copy number aberration, MSI: microsatellite instable, MSS: microsatellite stable, SNV: single nucleotide variant. (PDF 830 k [file 12943_2017_691_MOESM2_ESM.pdf]

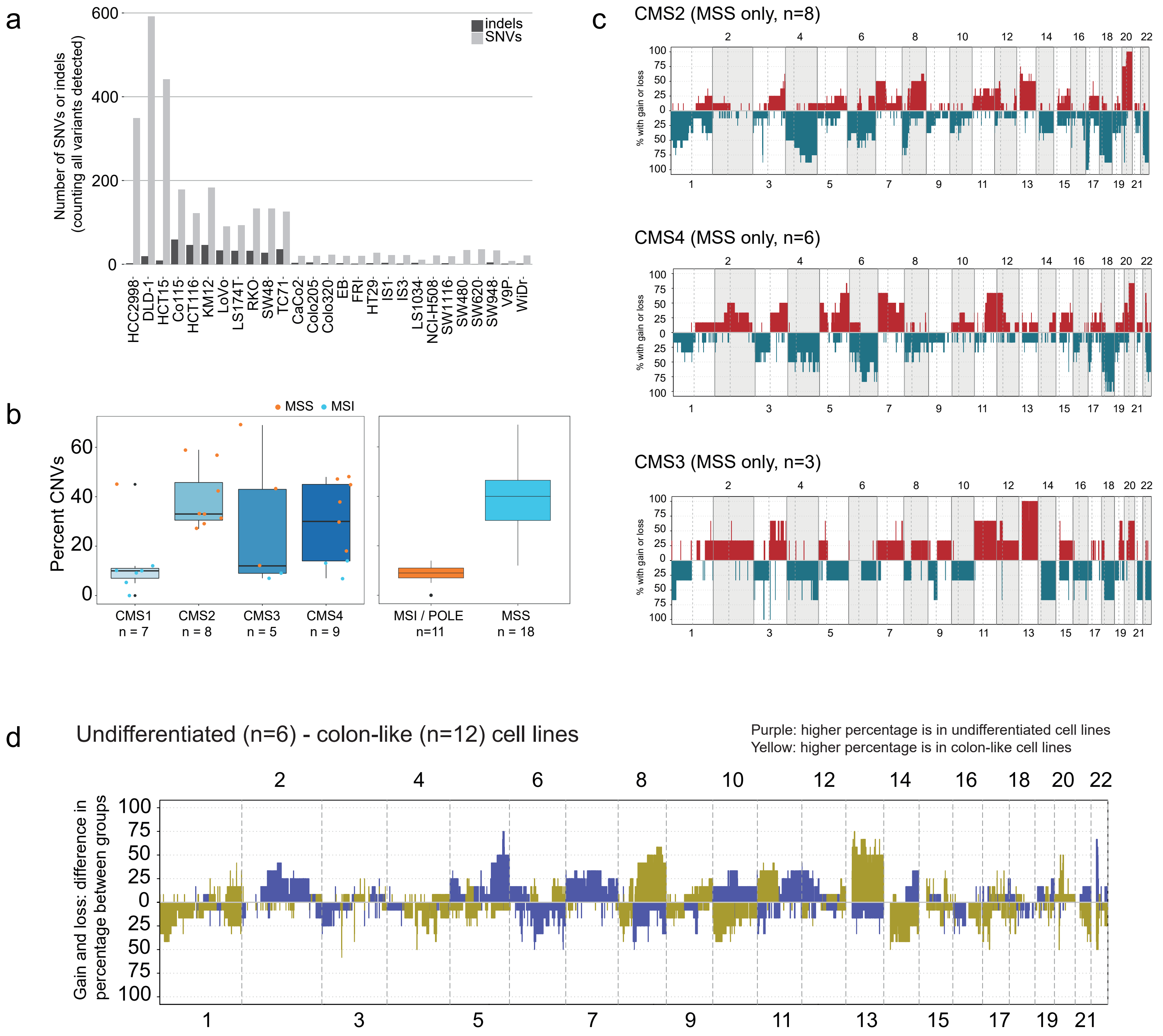

DNA level aberrations. (a) SNVs and indel counts in 34 cell lines. MSI cell lines generally displayed numerous SNVs/indels, in contrast to MSS cell lines, although DLD-1/HCT15 were less typical with a lower indel burden compared to remaining MSIs. (b) The percentage of the genome with aberrant CNA reflects MSI status rather than CMS subtype. The figure includes 29 unique MSI/MSS cell lines. (c) CMS frequency of CNAs. Vertical axis indicates frequency, horizontal axes shows chromosomes 1-22, separated by vertical lines (whole lines separates chromosomes, dashed lines separates chromosome arms). The most common gains in CMS2 (5 or more out of 9 CMS2 MSI/MSS cell lines) were found on 3q, 8q, 13q, 17q, 20p and 20q, while regions of loss were frequent on 1p, 3p, 4q, 6p, 6q, 8p, 16p, 16q, 17p, 18 p, 18q, 20p and 22q. In CMS4 the most common gains (4 or more out of 7 CMS4 MSI/MSS cell lines) were found on 3q, 5p, 5q, 7p, 7q, 12p, 20p, 20q and 22q, while losses were frequent on 3p, 4p, 4q, 6q, 15q, 17p, 18q and 22q. The plots for CMS2 and CMS4 are placed together for easier visual comparison. A frequency plot for CMS3 was included, but the low sample number limits interpretations of frequent alterations in this group. (d) Differential frequencies of CNAs in undifferentiated versus colon-like cell lines. The vertical axis indicates the frequency difference between undifferentiated – colon-like cell lines (i.e. the frequency in undifferentiated cell lines minus the frequency of aberration in colon-like cell lines). The horizontal axis indicates chromosomes 1-22 (chromosomes separated by whole lines, chromosome arms separated by dashed lines). Yellow areas represent regions with higher frequencies of CNAs in colon-like cell lines, purple areas represent regions with higher frequencies of CNAs in undifferentiated cell lines. CMS: consensus molecular subtype, CNA: copy number aberration, MSI: microsatellite instable, MSS: microsatellite stable, SNV: single nucleotide variant.
