# Supplementary material for: Multi-omics of 34 colorectal cancer cell lines - a resource for biomedical studies
Source: Mol Cancer. 2017 Jul 6;16:116. doi: 10.1186/s12943-017-0691-y (PMC5498998; doi:10.1186/s12943-017-0691-y)
Supplement: Supplementary file 3 — Expression differences between colon-like and undifferentiated cell lines. a PCA plots show the spontaneous split between the two subgroups in all three datasets (mRNA, miRNA and protein). b Volcano plots show differentially expressed genes in undifferentiated (blue) versus colon-like (yellow) cell lines on the mRNA, miRNA and protein levels. Horizontal dashed lines mark the highest p-value that produces an adjusted p-value of <0.01. Vertical dashed lines mark log2 fold-change (1 for mRNA/miRNA, 0.1 for protein). The top five differentially expressed mRNA/miRNA/proteins in terms of log2 fold-change within these thresholds are indicated by names, and the rest by filled circles. PCA: principal component analysis, PC1: principal component 1, PC2: principal component 2. (PDF 1095 kb) [file 12943_2017_691_MOESM3_ESM.pdf]

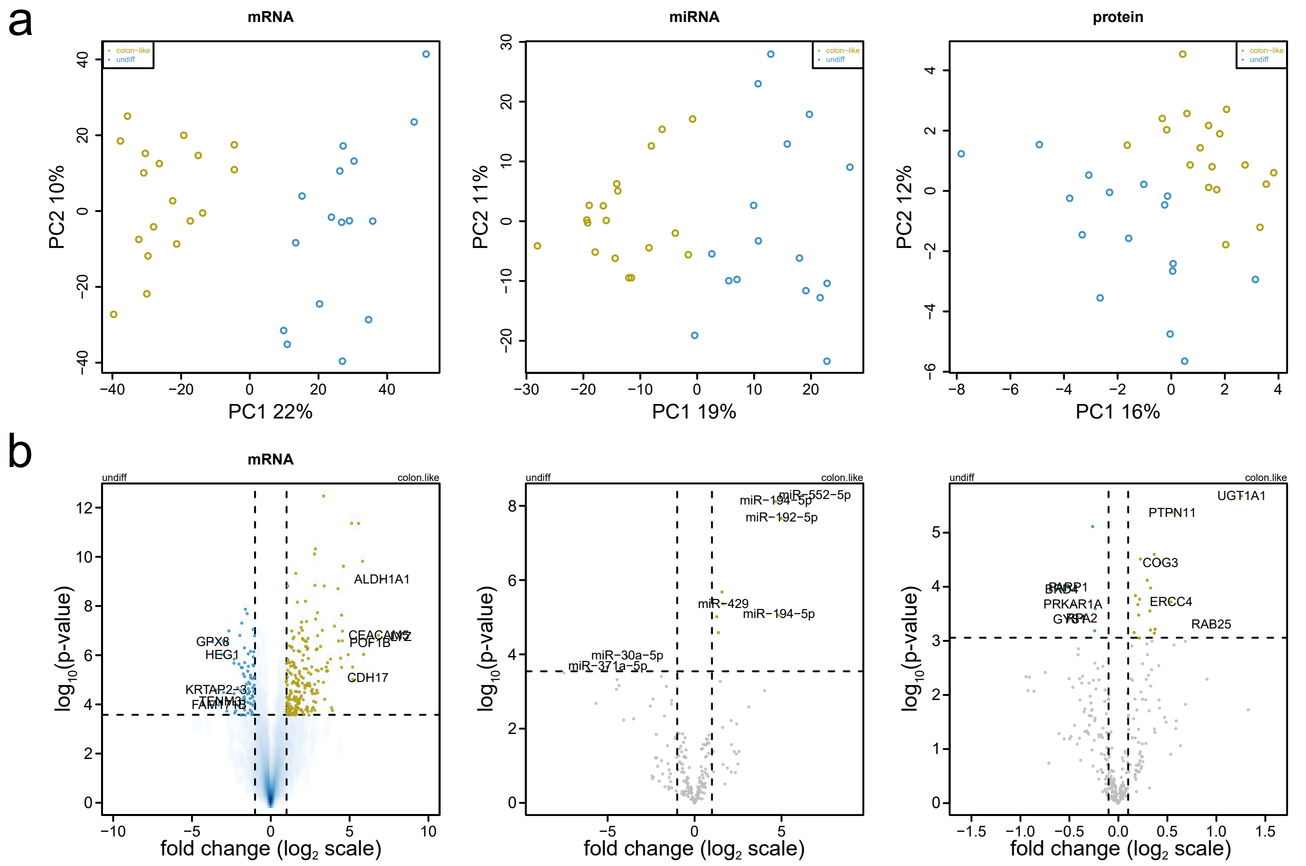

Figure S2: Expression differences between colon-like and undifferentiated cell lines. (a) PCA plots show the spontaneous split between the two subgroups in all three datasets (mRNA, miRNA and protein). (b) Volcano plots show differentially expressed genes in undifferentiated (blue) versus colon-like (yellow) cell lines on the mRNA, miRNA and protein levels. Horizontal dashed lines mark the highest p-value that produces an adjusted p-value of <0.01. Vertical dashed lines mark log2 fold-change (1 for mRNA/miRNA, 0.1 for protein). The top five differentially expressed mRNA/miRNA/proteins in terms of log2 fold-change within these thresholds are indicated by names, and the rest by filled circles. PCA: principal component analysis, PC1: principal component 1, PC2: principal component 2.
