# Supplementary material for: Multi-omics of 34 colorectal cancer cell lines - a resource for biomedical studies
Source: Mol Cancer. 2017 Jul 6;16:116. doi: 10.1186/s12943-017-0691-y (PMC5498998; doi:10.1186/s12943-017-0691-y)
Supplement: Supplementary file 4 — Outlier analysis reveals high level amplification events associated with marked mRNA expression changes, pointing to potential driver genes in individual cell lines. a A total of 280 genes were nominated, figure shows 15 nominated genes overlapping with the COSMIC Cancer Gene Census. Vertical axis shows gene expression values (log2 scale) and horizontal axis shows copy number aberration values (PCF values, log2 scale). The analysis pinpointed CNA values that were substantially higher in one or a few cell lines compared to remaining cell lines and hence 14 of the nominated genes were high amplification events, while one gene (SS18) was nominated on basis of all samples having loss except V9P. b Ten outlier genes had available associated protein data and for most genes the increase in gene expression was consistent on the protein level. Vertical axis shows relative protein expression (median centered normalized log2 values), horizontal axis shows gene expression values (log2 scale). (PDF 1233 kb) [file 12943_2017_691_MOESM4_ESM.pdf]

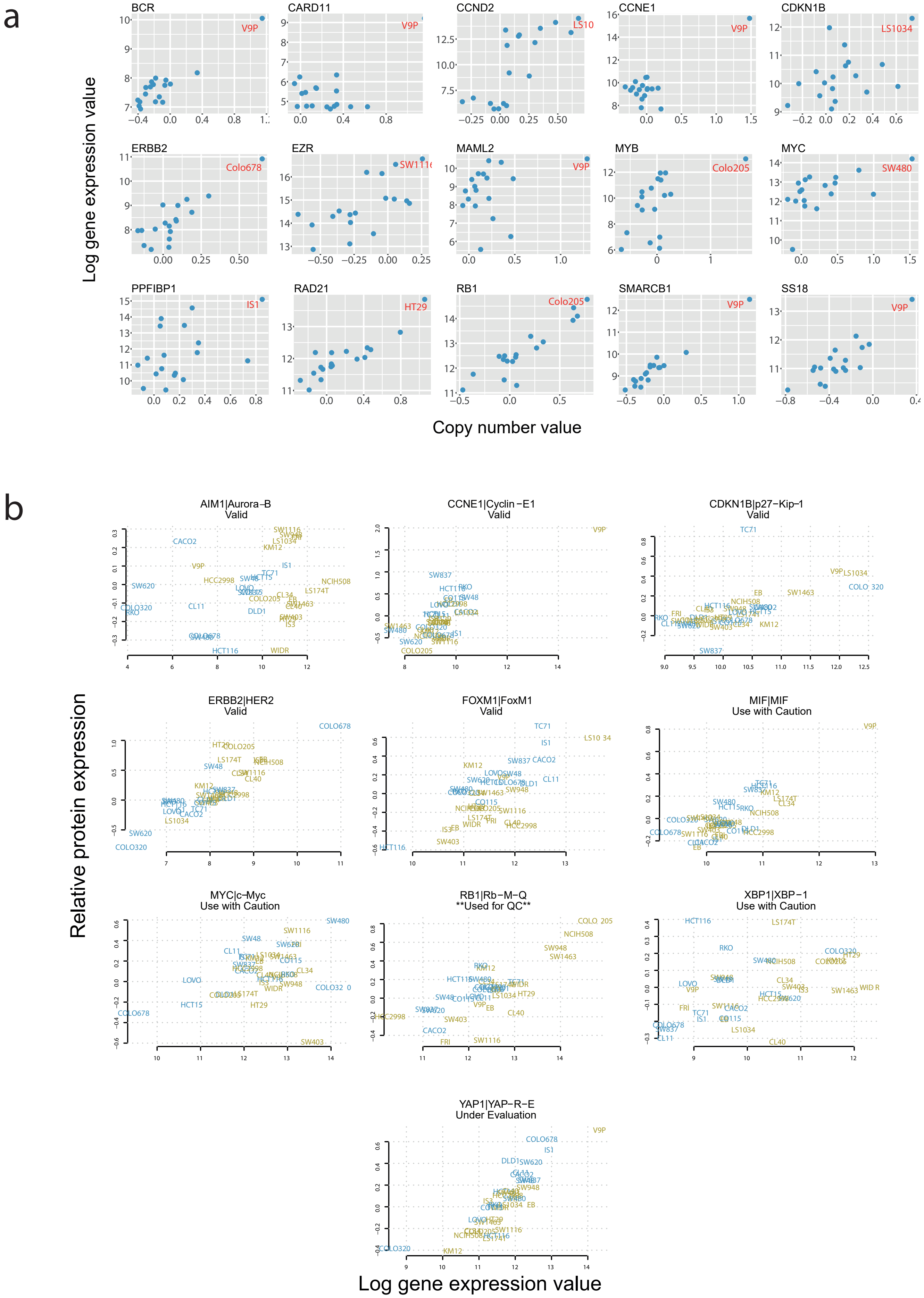

Figure S3: Outlier analysis reveals high level amplification events associated with marked mRNA expression changes, pointing to potential driver genes in individual cell lines. (a) A total of 280 genes were nominated, figure shows 15 nominated genes overlapping with the COSMIC Cancer Gene Census. Vertical axis shows gene expression values (log_2 scale) and horizontal axis shows copy number aberration values (PCF values, log_2 scale). The analysis pinpointed CNA values that were substantially higher in one or a few cell lines compared to remaining cell lines and hence 14 of the nominated genes were high amplification events, while one gene (SS18) was nominated on basis of all samples having loss except V9P. (b) Ten outlier genes had available associated protein data and for most genes the increase in gene expression was consistent on the protein level. Vertical axis shows relative protein expression (median centered normalized log_2 values), horizontal axis shows gene expression values (log_2 scale).
